# Effects of Muscle Strengthening around the Hip on Pain, Physical Function, and Gait in Elderly Patients with Total Knee Arthroplasty: A Randomized Controlled Trial

**DOI:** 10.3390/healthcare8040489

**Published:** 2020-11-17

**Authors:** KwangSun Do, JongEun Yim

**Affiliations:** 1 Department of Physical Therapy, Graduate School of Sahmyook University, Seoul 01795, Korea; kwangsun4988@gmail.com; 2 Department of Physical Therapy, Catholic Kwandong University International St. Mary’s Hospital, Incheon 22711, Korea; 3 Active Aging Research Institute, Sahmyook University, Seoul 01795, Korea

**Keywords:** total knee arthroplasty, hip muscle exercise, physical function, quadriceps, gait pattern

## Abstract

*Background*: Functional limitations may still remain even after a patient completes a traditional quadriceps-based rehabilitative program after total knee arthroplasty. Based on studies reporting that patients with knee osteoarthritis have muscle weakness around the hip joint after total knee arthroplasty, we investigated whether strengthening the hip muscles can reduce pain and improve the physical function and gait of patients who underwent total knee arthroplasty. *Methods*: Patients were randomly divided into three groups: hip, quadriceps, and control. The hip group (*n* = 19) completed an extensor, adductor, and external muscle strengthening exercise program. The quadriceps group (*n* = 20) completed a quadriceps strengthening exercise program. The control group (*n* = 16) completed an active range of motion exercises. Therapy was conducted thrice weekly for 12 weeks. Pain and function items from the Western Ontario and McMaster Universities Osteoarthritis Index, Alternate Step Test, Five Times Sit to Stand Test, and Single Leg Stance Test were performed to assess pain and physical function. In the gait analysis, stride, single-stance (%), double-stance (%), and gait speed were measured. Data were collected at baseline and at 4, 8, and 12 weeks after the intervention. *Results*: The hip group showed more significant improvements in pain and performance on the Alternate Step Test and Single Leg Stance Test than the quadriceps and control groups. In the gait analysis, the hip group showed the largest improvements in single stance and double stance. *Conclusions*: In conclusion, a 12-week hip muscle strengthening exercise program effectively improves the physical function and gait of patients who have undergone total knee arthroplasty.

## 1. Introduction

Osteoarthritis (OA) is a chronic, local joint disease that affects approximately a third of all adults, and its prevalence increases with age [1]. Most patients with OA undergo total knee arthroplasty (TKA) to reduce the pain and disability associated with knee OA. In South Korea, the number of cases of TKA increased by 28% from 2012 to 2016 [2]. TKA associated with OA causes pain and reduces functional independence and quality of life.

Evidence shows that the significant reduction in quadriceps strength following TKA negatively affects physical function [3,4]. For this reason, most rehabilitative protocols focus on the voluntary activation and muscle strength restoration of the quadriceps. However, a study reported that, while a traditional rehabilitative program reduced pain in patients who underwent TKA, the patients still had physical limitations one year after TKA [5]. Studies have reported that patients who underwent TKA have a 15% reduction in gait speed, 50% reduction in stair walk speed, 7–40% increase in fall risk, and 20% reduction in performance on the Six-Minute Walk Test compared to healthy subjects [3,6]. The functional limitations of patients with TKA indicate the need to expand the focus of current rehabilitation practices in order to restore the patient’s function to a healthy adult level.

Evidence shows the muscles around the hip joint are weakened in patients with knee OA [7]. Patients with knee OA adopt gait patterns that do not cause pain or minimize the load applied to the affected cartilages [8]. Such patterns result in a reduced gait speed, reduced stride length, and increased double-stance duration [9]. Reduced physical activities associated with these factors reduce the activity and muscle strength of the hip muscles [10]. The weakening of the hip muscles, which play an important role in stabilizing the pelvis, can apply an inappropriate loading on the knees. Recent studies have reported that the adductors and abductors of the hip joint play an important role in reducing an inappropriate moment arm on the knees [11,12,13,14]. It is important to improve the static stability of the muscles of the proximal limbs to prevent and treat the pathological changes in the muscles of the distal limbs [15,16]. The muscles of the proximal limbs can stabilize the hip joint and control the center of gravity of the body to change the load on the knee joints during walking. Studies reported that hip muscle strengthening exercise programs reduced pain and improved the physical functions of patients with end-stage knee OA [16,17].

Unfortunately, evidence shows that exercise programs that focus on the quadriceps do not strengthen the hip muscles of patients with TKA [18,19,20]. Pain, reduced usage of the hip muscles, and gait patterns adopted after surgery can result in the additional weakening of the hip muscles [18,20]. Given that the hip muscle strength can affect knee joint loads and physical functions, hip muscle strengthening may be considered as a new method of intervention for the rehabilitation of patients who have undergone TKA. However, the evidence is lacking regarding the therapeutic effect of hip muscle strengthening exercises on patients who underwent TKA. Therefore, this study investigated the effects of a quadriceps-based traditional rehabilitative program and hip muscle strengthening exercise program on the pain and physical functions of patients who have undergone TKA. We further hypothesized that a hip exercise program would improve knee ROM (Range of Motion), pain, physical function, and gait. The results of this study would be evidence supporting the benefits of additional hip exercise in ROM, pain, physical function, and gait.

## 2. Materials and Methods

### 2.1. Trial Design

This study is an assessor- and single-blinded randomized controlled trial (RCT). The hip group (Group A) was assigned an active range of motion exercises and hip muscle strengthening exercises. The quadriceps group (Group B) was assigned an active range of motion exercises and quadriceps strengthening exercises. The control group (Group C) was assigned an active range of motion exercises. This RCT was conducted in compliance with the CONSORT statement for reporting non-pharmacological interventions. This study was approved by the Sahmyook University Institutional Review Board (2-7001793-AB-N-012018099HR). The research protocol was registered with the Korea clinical trial registry (http://clinicaltrials.gov, CRIS identifier: KCT0004677).

### 2.2. Randomization and Allocation Concealment

**Figure 1:**
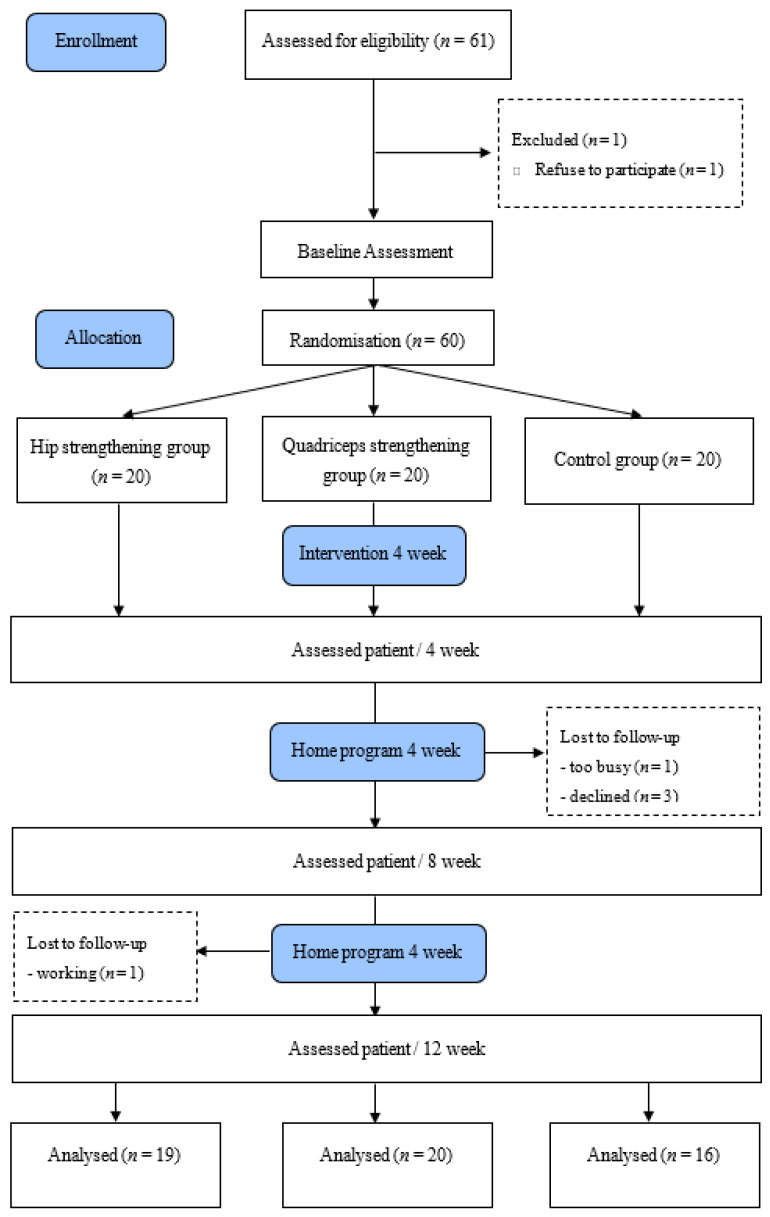
Flow diagram of the experimental procedure.

Patients were randomly assigned to a group by an assessor who did not perform a treatment or examination using the Graphpad software version 7.00 (http://www.graphpad.com/quickcalcs/randomize1/). A blinded assessor obtained measurements at the baseline (Week 0), Week 4, Week 8, and Week 12. For patients who underwent bilateral TKA, measurements were obtained from the knee in more severe conditions (Figure 1).

### 2.3. Procedure

Participants were interviewed through phone calls before the start of the study and provided with written information about the study after being screened by a physiotherapist. After consenting to participate, the participants were randomly assigned to one of three treatment groups. All the assessments were conducted by a blinded assessor, who was a physiotherapist. Measurements were obtained at a place different from the place of the intervention to prevent the physiotherapist responsible for observing and providing the intervention from observing the assessments. The physiotherapist who provided the intervention and obtained measurements had 5–10 years of experience. Group A and Group B participated in a four-week exercise program to receive education from the physiotherapist on exercises, monitoring, and how to safely complete resistance training. They then participated in a home exercise program from Week 5 to Week 12. Exercise logs and weekly phone call interviews were used to check whether the participants properly finished their exercises.

### 2.4. Participants

Of patients aged 59~90 years who underwent TKA due to knee OA within three months to one year ago, those who had pain scores of four points or higher on the visual analogue scale (0 = no pain, 10 = maximum pain) for a week or longer could participate in this study. The exclusion criteria included: (1) unstable medical condition, such as uncontrolled cardiovascular disease or uncontrolled diabetes; (2) neurological or any other conditions affecting the strength or function of the lower limbs; (3) being diagnosed with a serious disease that can cause knee pain; and (4) ipsilateral hip osteoarthritis or lateral hip pain.

### 2.5. Intervention

The hip group and quadriceps group attended three exercise sessions per week (one session was home exercise) for four weeks. After completing the four-week training, the participants performed home exercises from Week 5 to Week 12. The exercises they performed are described in the tables below (Table 1 and Table 2).

#### 2.5.1. Hip Muscle Group (A)

The participants performed four exercises designed to strengthen the hip abductor, hip external rotator, hip adductor, and hip extensor muscles (Table 2). The participants were provided with proper education on exercise and monitored. The participants performed exercises by visiting a physical therapist twice a week for four weeks after the basic assessment and performed an additional home exercise once for 4 weeks. The participants were given a 1–2-min break between each set. Once the four-week training was over, the participants switched to home exercises and were reached via weekly phone calls to check if they had completed all the assigned exercises.

#### 2.5.2. Quadriceps Muscle Group (B)

The participants performed three exercises designed to strengthen the quadriceps. The details of the exercises are the same as those for the hip group (Table 2).

#### 2.5.3. Control Group (C)

The control group performed AROM (Active Range of Motion) of the knees for 12 weeks.

### 2.6. Outcome Measure

#### 2.6.1. Range of Motion (ROM)

Active knee flexion and extension ROM were measured in the supine position with a long axis goniometer [21]. Positive values indicate positions of knee flexion and negative values indicate positions of knee hyperextension [22].

#### 2.6.2. Alternative Step Test (AST)

The Alternate Step Test requires speed, strength, and balance, as it involves placing the whole foot onto a step which is 18 cm high and 40 cm deep and alternating with the right and left feet, for a total of eight repetitions as quickly as possible. The time taken to complete the task is the score [23].

#### 2.6.3. Five Times Sit to Stand Test (FTSST)

A FTSST was performed by each participant to quantify the performance of transitions between sitting and standing [24]. At the start of this test, the participants were seated in a chair of standardized seat height (46 cm) with their feet placed on the center of each force platform in a participant-selected degree of comfortable knee flexion. The time taken to transition between the sitting and standing positions 5 times as quickly as possible was recorded. The start point and endpoint of the test were the participants being seated with his/her back touching the backrest of the chair.

#### 2.6.4. Times up and Go (TUG)

The TUG Test measures the time, in seconds, that a patient takes to stand from an armed chair, walk for 3 m, and return to sit on the same chair. A walking aid can be used if required [25]. The subjects were allowed to use the armrests and were wearing their shoes.

#### 2.6.5. Six Meter Walking Test (6MWT)

The 6 min walk test (6MWT) is a frequently used measure for patients following TKA [26,27,28,29,30]. It is a test of aerobic capacity and long-distance walking ability [29]. Patients are instructed to walk with their usual gait aids on a premeasured circuit, covering as much distance as possible during the 6 min time frame. Rests are permitted and are included in the time.

#### 2.6.6. Single Leg Stance (SLS)

The SLS Test was a measure of balance that consisted of recording the length of time the participants balanced on one leg while keeping their hands on their hips. The test lasted up to 30 s and was stopped if: (1) the swing leg touched the floor, (2) the tested foot displaced on the floor, (3) the swing lower leg touched the tested limb, or (4) the arms swung away from the hips. These tests cover important domains of lower-extremity physical function, such as walking ability, dynamic and static balance, muscle strength and power, and movement control [31].

#### 2.6.7. Gait Analysis

Gait parameters were assessed with the OptoGait (Microgate Co, Bolzano, Italy). The OptoGait is a 3 m walkway designed for optical-sensitive analysis. The subjects completed a single trial at free speed with the instruction to “walk at your normal speed”. Individually determined rest periods were provided between the single trials undertaken. The gait speed and mean step length were calculated using specific software (OptoGait analysis software, version 1.6.4.0).

#### 2.6.8. Self-Reported Symptoms and Functional Status

The 24-item WOMAC (Western Ontario and McMaster Universities Osteoarthritis Index) was used to gather knee-specific information on symptoms and functional limitations. The WOMAC is a valid and reliable disease-specific measure of pain, stiffness, and physical function in patients with knee OA [32,33]. Higher scores on the WOMAC subscales indicate an increased severity of symptoms or functional limitations. The pain and physical function items of the subscale were used in this study.

### 2.7. Sample Size Calculations

The repeated measures analysis of variance was set at a significance level (α) = 0.05, effect size f = 0.25, and power = 0.95; this required a sample size of 51 patients to maintain an actual power of 0.96. To account for patients who would discontinue treatment (drop-outs), we estimated that a total of 61 patients would be required.

### 2.8. Data and Statistical Analysis

IBM SPSS Statistics version 22.0 software for Windows (IBM Corp, Armonk, NY, USA) was used for statistical processing in this study. The characteristics of the subjects were analyzed using a chi-square test and descriptive statistics. The Shapiro–Wilk test was used for the normality testing of the participants’ general characteristics. A one-way analysis of variance (ANOVA) was performed to test the homogeneity of the dependent variables between the three groups at the baseline. Additionally, a mixed-model analysis was applied to compare the obtained values of the quantitative variables of the three study groups (A group, B group, and C group) and at the four measurement times (baseline, 4 weeks, 8 weeks, and 12 weeks). Data were analyzed using an analysis of covariance (ANCOVA), in which groups were compared according to baseline scores as the covariate. Bonferroni’s method was used for post-hoc testing. A statistical significance level of α = 0.05 was used for all tests.

## 3. Results

Sixty-one patients who underwent TKA were evaluated on their eligibility for study participation. Sixty participants (50 females and 10 males) consented to participate and were randomly assigned to one of three treatment groups (Figure 1). The three groups shared similar general characteristics, as shown in Table 3. During the research period, five participants dropped out due to personal reasons, and the remaining 55 patients (47 females and 8 males) completed their study participation (Table 3). The general characteristics of the subjects did not differ significantly among the A group, B group, or C group. The participants completed exercise sessions three times a week.

### 3.1. Pain and Range of Motion

RM-ANOVA shows a significant “time × group” interaction effect for each range of motion and pain (*p* < 0.05). In a post-hoc analysis, Group A and Group B showed significant differences in flexion angles compared to Group C. Significant differences in WOMAC-Pain and WOMAC-Function scores were found between Group A and Group C (Table 4).

### 3.2. Physical Function

RM-ANOVA shows a significant “time × group” interaction effect for AST, FTSST, TUG, 6MWT, and SLS (*p* < 0.05). In a post-hoc analysis, Group A and Group B showed significant differences in the FTSST and TUG Test scores compared to Group C. Group A showed significant improvements in the AST, 6MWT, and SLS scores compared to Group B and Group C (Table 5).

### 3.3. Gait Analysis

RM-ANOVA shows a significant “time × group” interaction effect for single stance, double stance, and speed (*p* < 0.05). In a post-hoc analysis, Group A showed significant improvements on single stance and double stance compared to Group B and Group C. Group A and Group B had significantly higher gait speeds than Group C (Table 6).

## 4. Discussion

This single-blind randomized controlled trial investigated the effect of a 12-week hip muscle strengthening exercise program on the physical function and gait of patients who underwent TKA within three months to one year. Quadriceps are often strengthened to reduce knee pain and improve the physical function in patients with OA or patients who underwent TKA [34,35]. Quadriceps strengthening exercises are also commonly included in home exercise programs to continuously manage treatment outcomes and for their economic benefits. Many researchers have been focusing on knee problems to improve the physical function of patients who underwent TKA [36,37]. Despite their effort, patients who undergo TKA have weakened lower limbs and continue to have functional limitations such as slow gait speed and difficulty climbing the stairs compared to healthy subjects [4,38,39,40,41]. A previous study reported that strengthening the hip muscles closer to the proximal limbs instead of strengthening the muscles around the source of pain reduced the pain and improved physical functions of patients with knee OA [16,33], suggesting that the hip muscles, which are closer to the proximal limbs than are the knees, can contribute to the mechanical changes in the knee joints. Since the muscles around the hip play an important role in stabilizing the pelvis and trunk, they can affect the moment arm of the knees [11]. Therefore, it is necessary to strengthen not only the knees but also the muscles around the hip through muscle strengthening exercises to improve pain, physical function, and gait after TKA.

This study demonstrated that hip muscle strengthening exercises significantly affect pain, physical function, and gait. The pain item of the WOMAC questionnaire was used to assess pain levels. All three groups showed improvements in pain levels over time. Group A showed a significant improvement in pain levels compared to the control group. This result was consistent with a previous report showing that a hip muscle strengthening exercise program reduces knee pain in patients with OA [16,42]. Based on these results, hip muscle strengthening exercises may be a good alternative for patients who find quadriceps exercises too painful. From Week 5 to Week 12, during which the exercise program was switched to the home exercise program, the magnitude of reductions in pain levels decreased compared to the pain reductions observed during the 4-week period in which the participants exercised under the guidance of a physiotherapist (Table 4). In a previous study, a home exercise program reduced pain in patients with knee OA, but only a small reduction of 0.77 was found on the pain item of the WOMAC questionnaire [16]. This may be due to the inaccuracy of phone calls and exercise logs in monitoring whether the participants properly completed their exercises in a home exercising program, as compared to directly checking the participants during a therapy session. A remote video call system may be one solution to this problem.

A major finding from the physical function tests is that Group A showed significant improvements in the AST and SLS test scores compared to Group B and Group C. This finding supports previous reports that the lateral and medial muscles of the hip play an important role in controlling the pelvis and trunk [11,12,13,14,43]. Since patients had to stand on one leg in most of these tests, it appears that stabilizing the muscles that control the movement of the hip joint in the frontal plane had a greater impact on the pelvis and trunk stabilization compared to the knees in the sagittal plane. The hip and quadriceps groups showed improvements in other physical function tests including the FTSST, TUG Test, and 6MWT compared to the control group. In an RCT that compared the effect of hip muscle strengthening exercises and that of quadriceps strengthening exercises on patients with knee OA, both exercises improved patients’ performance on tests that require the strong extension of the knees such as the FTSST [42]. On the other hand, in the Climb Stairs Test, which requires a single-leg stance, hip muscle strengthening exercises resulted in higher improvements than quadriceps strengthening exercises.

Patients with OA have shown a reduced gait speed, reduced stride length, shorter single-stance phase, and longer double-stance phase before a surgical intervention [4,44,45,46,47]. A previous study reported that patients with OA walk slowly and never recover normal gait patterns even after a successful operation. Consequently, pain relief has become the main goal of surgery for patients with OA. The major findings of our gait analysis are that Group A showed a longer single-stance leg phase and a shorter double-stance phase compared to Group B and Group C. The hip muscle strengthening exercises not only improve the single stance-based physical function but also play an important role in restoring normal gait patterns.

Our study has a few limitations. First, since we did not compare the strengths of the hip muscles and quadriceps with those of a healthy population, we could not examine by how much the muscle strengths were reduced in our sample population. Second, limited quantification and comparisons were conducted in our analysis, since the participants could not be followed up for a long term.

## 5. Conclusions

In summary, a 12-week hip muscle strengthening exercise program improved the pain, physical function, and gait patterns of patients who underwent TKA. The program especially effectively improved single stance-based function. A rehabilitative program for patients who underwent TKA developed in consideration of these benefits of hip muscle strengthening exercise may effectively promote the recovery of the patients. Therefore, to improve single stance-based physical function and gait in patients with TKA, additional hip exercises combined with conventional quadriceps exercises are required.

## Figures and Tables

**Table 1:** Hip abductor and adductor muscle strengthening exercises.

Exercise	Dosage
Warm up (AROM)	10 min
Supine extension bridge with thera-band	3 sets of 20 at a RPE 5~7
Sideway walking with thera-band	3 sets of 20 steps at a RPE 5~7
Standing hip adduction with thera-band	3 sets of 20 at a RPE 5~7
Clamshell (Hip external rotation) with thera-band	3 sets of 20 at a RPE 5~7

**Table 2:** Quadriceps muscle strengthening exercises.

Exercise	Dosage
Warm up (AROM)	10 min
Seated knee extension with thera-band	4 sets of 20 at a RPE 5~7
Supine straight leg raise with thera-band	4 sets of 20 at a RPE 5~7
Quarter wall squat with thera-band	4 sets of 20 at a RPE 5~7

**Table 3:** General characteristics of participants (*n* = 55).

Characteristic	All Patients	Hip Group	Quadriceps Group	Control Group	*p* Value
	Mean (SD)	Mean (SD)	Mean (SD)	Mean (SD)	
Sex (Men/Women)	8(16.4%)/47(83.6%)	3(15.8%)/16(84.2%)	3(15%)/17(85%)	3(18.8%)/13(81.3%)	0.952 (chi-Square test)
Age (years)	72.80 ± 5.47	72.84 ± 7.03	72.50 ± 4.73	73.13	0.945
Height (cm)	154.76 ± 5.64	155.07 ± 5.86	154.28 ± 5.25	155.00 ± 6.17	0.894
Weight (kg/m^2^)	60.65 ± 5.00	60.58 ± 5.02	61.05 ± 5.50	60.25 ± 4.61	0.893
BMI (kg/m2)	37.47 ± 2.76	37.19 ± 2.59	37.60 ± 3.03	37.62 ± 2.75	0.868

SD, standard deviation; BMI, body mass index; ROM, range of motion; WOMAC, Western Ontario and McMaster Universities Osteoarthritis Index.

**Table 4:** Outcome scores for each group at baseline (week 0), post-intervention (week 4), follow-up (week 8), and follow-up 2 (week 12): mean and standard deviation.

Outcome Measure	Hip Group	Quadriceps Group	Control	F	*p*	Post Hoc
	Mean (SD)	Mean (SD)	Mean (SD)			
Flexion ROM						
Baseline	114.42 ± 10.52	115.15 ± 9.33	117 ± 5.79	0.378	0.687	
4 week f/w	118.32 ± 9.39	120.4 ± 7.25	118.44 ± 5.66	5.8	0.005 *	B > C
8 week f/w	119.53 ± 9.19	121.45 ± 6.73	118.56 ± 5.64	9.274	0.001 *	A, B > C
12 week f/w	119.84 ± 8.82	122.7 ± 6.4	118.88 ± 5.62	11.006	0.001 *	A, B > C
Time × Group				3.382	0.004 *	
Extension ROM						
Baseline	10.37 ± 5.6	8.1 ± 3.58	9.13 ± 3.09	1.371	0.263	
4 week f/w	8.58 ± 4.76	6.8 ± 2.72	8.19 ± 2.56	1.509	0.231	
8 week f/w	7.79 ± 4.41	6.2 ± 2.3	8 ± 2.65	4.776	0.013 *	A, B > C
12 week f/w	7.84 ± 4.11	6.1 ± 2.33	7.63 ± 2.6	2.604	0.084	
Time × Group				2.291	0.041 *	
WOMAC-P						
Baseline	12.32 ± 2.42	12.75 ± 2.78	12.19 ± 2.56	0.239	0.788	
4 week f/w	8.21 ± 4.14	10.1 ± 2.97	11.25 ± 1.91	6.547	0.003 *	A > C
8 week f/w	8.37 ± 2.56	9.6 ± 2.41	11 ± 2.12	11.474	0.001 *	A, B > C
12 week f/w	8.05 ± 3.2	9.8 ± 2.39	10.69 ± 2.15	7.677	0.001 *	A > C
Time × Group				3.493	0.004 *	
WOMAC-F						
Baseline	41.89 ± 5.36	45.1 ± 7.13	45.31 ± 4.78	1.931	0.155	
4 week f/w	33.74 ± 8.93	38.2 ± 6.38	43.56 ± 3.75	8.464	0.001 *	A, B > C
8 week f/w	33.32 ± 8.49	37.35 ± 5.94	41.5 ± 4.32	5.767	0.006 *	A > C
12 week f/w	32.05 ± 8.76	36.75 ± 5.32	40.31 ± 4.84	5.499	0.007 *	A > C
Time × Group				1.456	0.201	

SD, standard deviation; ROM, range of motion; WOMAC, Western Ontario and McMaster Universities Osteoarthritis Index. * *p* < 0.05.

**Table 5:** Outcome scores for each group at baseline (week 0), post-intervention (week 4), follow-up (week 8), and follow-up 2 (week 12): mean and standard deviation.

Outcome Measure	Hip	Quadriceps	Control	F	*p*	Post Hoc
	Mean (SD)	Mean (SD)	Mean (SD)			
AST						
Baseline	15.72 ± 3.98	16.13 ± 4.99	14.24 ± 3.18	0.977	0.383	
4 week f/w	12.17 ± 2.66	14.26 ± 3.54	13.76 ± 2.85	7.684	0.001 *	A > B, C
8 week f/w	11.68 ± 3.3	14.31 ± 3.53	13.67 ± 2.51	10.470	0.001 *	A > B, C
12 week f/w	11.17 ± 2.69	14.46 ± 3.8	13.23 ± 2.6	13.336	0.001 *	A > B, C
Time × Group				3.999	0.001 *	
FTSST						
Baseline	15.25 ± 3.16	16.25 ± 3.64	14.49 ± 2.8	1.326	0.274	
4 week f/w	13.25 ± 2.6	13.12 ± 2.76	13.92 ± 2.29	13.595	0.001 *	A, B > C
8 week f/w	12.58 ± 2.58	12.77 ± 1.99	13.79 ± 2.01	11.074	0.001 *	A, B > C
12 week f/w	12.69 ± 2.7	13.26 ± 2.32	13.29 ± 1.97	4.09	0.022 *	A, B > C
Time × Group				4.699	0.001 *	
TUG						
Baseline	14.19 ± 2.37	15.44 ± 3.13	15.38 ± 3.1	1.122	0.333	
4 week f/w	11.09 ± 2.29	12.7 ± 2.79	14.76 ± 2.27	16.737	0.001 *	A, B > C
8 week f/w	10.68 ± 2.36	12.46 ± 2.74	14.52 ± 2.41	16.769	0.001 *	A, B > C
12 week f/w	10.34 ± 2.3	12.31 ± 2.63	14.47 ± 2.27	24.661	0.001 *	A, B > C
Time × Group				4.394	0.001 *	
6MWT						
Baseline	285.54 ± 98.57	244.54 ± 93.71	240.31 ± 51.72	1.568	0.218	
4 week f/w	341.81 ± 109.11	295.55 ± 86.63 *	245.43 ± 50.22	9.994	0.001 *	A, B > C
8 week f/w	351.91 ± 100.6	289.1 ± 89.3 *	247.62 ± 52.86	6.74	0.002 *	A, B > C
12 week f/w	346.67 ± 101.95	284.6 ± 87.04 *	252.93 ± 51.9 *	5.67	0.006 *	A > C
Time × Group				4.209	0.001 *	
SLS						
Baseline	5.68 ± 2.51	7.37 ± 1.95	6.48 ± 2.93	2.312	0.109	
4 week f/w	8.56 ± 3.16	8.08 ± 1.89	6.56 ± 2.94	14.374	0.001 *	A > B, C
8 week f/w	9.83 ± 3.57	8.15 ± 1.94	6.32 ± 2.94	11.817	0.001 *	A > B, C
12 week f/w	9.7 ± 3.82	7.85 ± 2.18	6.95 ± 2.93	7.837	0.001 *	A > B, C
Time × Group				8.381	0.001 *	

SD, standard deviation; AST, alternative step test; FTSST, five time sit to stand test; TUG, time up and go test; 6MWT, six meter walk test; SLS, single leg stance. * *p* < 0.05.

**Table 6:** Outcome scores for each group at baseline, post-treatment, 8 week follow-up, and 12 week follow-up: mean and standard deviation (gait analysis).

Outcome Measure	Hip	Quadriceps	Control	F	*p*	Post Hoc
	Mean (SD)	Mean (SD)	Mean (SD)			
Stride (cm)						
Baseline	87.94 ± 10.20	87.58 ± 13.45	85.37 ± 8.48	0.268	0.766	
4 week f/w	94.85 ± 13.00	90.65 ± 10.37	85.82 ± 8.82	24.768	0.024 *	A > C
8 week f/w	97.59 ± 13.14	90.85 ± 10.29	86.15 ± 8.52	5.902	0.005 *	A > C
12 week f/w	97.24 ± 10.46	90.70 ± 10.24	87.16 ± 8.94	7.304	0.002 *	A > B,C
Time × Group				1.730	0.122	
Single-stance(%)						
Baseline	31.71 ± 3.03	31.98 ± 2.30	31.64 ± 2.40	0.086	0.918	
4 week f/w	34.37 ± 3.21	32.67 ± 2.45	31.81 ± 2.48	10.225	0.001 *	A > B,C
8 week f/w	35.31 ± 3.78	32.44 ± 2.42	31.81 ± 2.58	12.574	0.001 *	A > B,C
12 week f/w	34.99 ± 3.36	32.60 ± 2.39	32.23 ± 2.31	12.155	0.001 *	A > B,C
Time × Group				3.933	0.001 *	
Double-stance(%)						
Baseline	33.37 ± 2.41	32.74 ± 4.28	33.69 ± 3.68	0.343	0.711	
4 week f/w	29.62 ± 3.61	31.76 ± 3.75	33.69 ± 3.38	12.702	0.001 *	A > B,C
8 week f/w	28.75 ± 4.32	31.88 ± 3.67	33.51 ± 3.22	11.999	0.001 *	A > B,C
12 week f/w	28.90 ± 3.53	31.98 ± 3.83	33.24 ± 3.14	14.983	0.001 *	A > B,C
Time × Group				4.462	0.001 *	
Speed (m/s)						
Baseline	0.82 ± 0.12	0.82 ± 0.11	0.80 ± 0.10	0.257	0.774	
4 week f/w	0.93 ± 0.11	0.93 ± 0.11	0.81 ± 0.09	10.619	0.001 *	A,B > C
8 week f/w	0.97 ± 0.11	0.96 ± 0.11	0.81 ± 0.09	18.006	0.001 *	A,B > C
12 week f/w	0.96 ± 0.11	0.98 ± 0.12	0.82 ± 0.09	14.179	0.001 *	A,B > C
Time × Group				4.270	0.001 *	

SD, standard deviation. * *p* < 0.05.
